# Weekly variations of accelerometer variables and workload of professional soccer players from different positions throughout a season

**DOI:** 10.1038/s41598-023-29793-5

**Published:** 2023-02-14

**Authors:** Hadi Nobari, Gibson Moreira Praça, Sarah da Glória Teles Bredt, Pablo Prieto González, Filipe Manuel Clemente, Jorge Carlos-Vivas, Luca Paolo Ardigò

**Affiliations:** 1 Department of Exercise Physiology, Faculty of Educational Sciences and Psychology, University of Mohaghegh Ardabili, Ardabil, Iran; 2 Department of Motor Performance, Faculty of Physical Education and Mountain Sports, Transilvania University of Braşov, Brasov, Romania; 3 Faculty of Sport Sciences, University of Extremadura, Cáceres, Spain; 4 Sports Scientist, Sepahan Football Club, Isfahan, Iran; 5 Sports Department, Universidade Federal de Minas Gerais, Belo Horizonte, Brazil; 6 Department of Physical Education, Prince Sultan University, Riyadh, Saudi Arabia; 7 Escola Superior Desporto e Lazer, Instituto Politécnico de Viana do Castelo, Rua Escola Industrial e Comercial de Nun’ Álvares, Viana do Castelo, Portugal; 8 Department of Teacher Education, NLA University College, Oslo, Norway; 9 Instituto de Telecomunicações, Delegação da Covilhã, 1049-001 Lisboa, Portugal

**Keywords:** Physiology, Health care

## Abstract

The current study aimed to analyze, using accelerometer-based activity, acute workload, chronic workload, acutechronic workloads ratio, training-monotony and training-strain throughout a competitive soccer-season and to compare these variables between players from different playing positions. Twenty-one professional soccer-players were monitored during the 48 weeks of the season. Players were grouped according to their position. Four lateral-defenders and four winger-players formed LDW group, four central-defenders and four forwards formed CDF group, and six midfielder-players formed MDF group. Accelerometer-based variables were collected during training and match contexts and were used to generate indicators of weekly acute and chronic workload, training monotony, training strain and metabolic power. A one-way ANOVA compared all dependent variables between groups, and effect sizes for pairwise comparisons were calculated. Results revealed variations in the weekly load throughout the season, which demands caution from coaches to avoid injuries. There were no differences in weekly-loads for all dependent variables (P > 0.05, small-to-moderate effects). We conclude that the weekly-load is not constant during a competitive season and players from different positions have similar weekly-loads. Therefore, previously reported in the literature, possible match-related positional differences might be compensated by differences in training-related loads, leading to a similar profile when considering the whole week.

## Introduction

Stimulating players with adequate training loads challenges coaches in elite soccer. Recent approaches to load monitoring proposed that variations in weekly training load reduce monotony and increase the potential for positive adaptations^1^. However, the competitive schedule requires frequent adjustments in load prescription because players are differently demanded during the matches^2,3^. For example, players from different playing positions differ in internal and external loads during a match^4,5^, leading to other weekly training loads. Hence, assessing training loads during matches and training is essential for an adequate load prescription.

The external training load is defined as the work performed by an athlete during training or competition^6,7^. Data recorded with Global Positioning Systems (GPS) allows coaches to calculate different variables associated with the external training load. Traditionally, external load data collection and recording has focused mainly on variables such as volume, intensity, and occasionally frenquency and density. However, since training load variability may condition the type of adaptations that can be attained, it is essential to consider parameters such as *training monotony, training strain*^1,3,8^, metabolic power, and acute-chronic workload ratio (ACWR)^9,10^, which were previously calculated using rates of perceived exertion only. *Training monotony* is the mean of the training loads performed during the week divided by the standard deviation of these training loads^11^; *training strain* is the sum of the training loads performed in all training sessions and matches during a week multiplied by training monotony^11^. The *metabolic power* is the overall energy cost of activity^12^, and the ACWR compares the acute workload (the workload players were exposed in the current week) with the chronic workload (i.e., the workloads players were exposed in the last weeks, usually^4,9,10^). Together, these variables describe the variation in the weekly training load and can be used to adjust the load prescription according to players’ responses. This multidimensional approach to load monitoring seems crucial in training^13^ and competition since isolated variables may not capture the whole phenomenon.

The impact of the playing position on match demands is well established. Bush et al.^4^ showed increased match demands among all playing positions in the past decade, mainly the high-intensity efforts. Full-backs and wing forwards perform the highest number of sprints during the match^14,15^, which may result in a higher external load in these players compared to the other playing positions. Hence, differences in the weekly training load in players from different playing positions are expected, although this hypothesis still needs to be tested. The limited knowledge about the possible differences in the weekly training loads in players from different playing positions limits the evidence-based load prescription and must be investigated.

Considering the abovementioned rationale, this study aimed to (1) analyze the acute and chronic training loads, the ACWR, the training monotony and the training strain throughout a competitive season in professional soccer players and (2) compare these variables between players from different playing positions. Furthermore, based on the match data, we expected that external players (e.g., lateral defenders and wingers) would present higher weekly training loads than the other playing positions.

## Methods

### Experimental approach to the problem

This study was a cohort of an entire season at the highest level of the Iranian Premier League, the Persian Gulf Premier League and the Hazfi cup in 2018–2019. In this study, based on the availability of one team, we divided of players into three groups based on similar activity needs of playing position^16^, so 4 Lateral Defender and 4 Wingers players formed the LDW group, another 4 Central Defender and 3 Forwards constituted the CDF group and 6 Midfielder players formed the MDF group. Players were monitored and controlled by GPS throughout the season (GPSPORTS, SPI high-performance unit).

The monitoring was performed daily for each training session and competition. Finally, we explored two specific goals, which are: (i) to describe (mean/standard deviation (SD)), weekly average acute (wAW), chronic (wCW), wACWR, training monotony (wTM) and training strain (wTS) based on body load (BL) variations across the full season by play position; (ii) to analyze the variations in pairwise comparisons by play position of wAW, wCW, wACWR, wTM, wTS, weekly metabolic power average (wMPA), weekly average of Accelerations zone 1 (wAccZ1), Accelerations zone 2 (wAccZ2), Accelerations zone 3 (wAccZ3), Decelerations zone 1 (wDecZ1), Decelerations zone 2 (wDecZ2), Decelerations zone 3 (wDecZ3), Ratio between wMPA/wAW, Ratio between wAccZ1/wDecZ1,Ratio between wAccZ2/wDecZ2, Ratio between wAccZ3/wDecZ3 in the full season.

### Participants

The study included 21 professional soccer players (28.3 ± 3.8 years; 181.2 ± 7.1 cm; 74.5 ± 7.7 kg; 22.6 ± 1.0 kg/m^2^) who were monitored for 48 weeks during a full season in the Iranian Premier League. For the players' information to be calculated, they had to attend at least three weekly sessions. The exclusion criteria of this study were defined as follows (i) If a player were absent for two consecutive weeks, he would be excluded from the study for analysis. (ii) Goalkeepers were not included in the study for analysis. The current study was approved by the University of Mohaghegh Ardabili (1395.10.20). As well as the club's official license and the players' informed consent were also obtained for study. We followed the Helsinki Declaration on Human Research at all stages of the study.

### Monitoring external load

#### GPS specifications

We used the GPSPORTS systems Pty Ltd, made in Australia, throughout the season to collect information for each training session and competition. According to the manufacturer's manual, this system includes the following specifications: (i) GPS with 15 Hz through the accelerometer variables (Acc & Dec); (ii) BL with 16G, 100 Hz, and Tri-Axial; (iii) All transfer of information is done by the infrared; (iv) It has the smallest size and is waterproof on the market; (v) It weighs about fifty grams and has very high battery power. Data collected during the season had good weather states regarding the satellite. GPS unit has high validity and reliability for measuring external load variables^17^.

#### Variables collected

To ensure accurate data collection by GPS, we pursued the following procedure. First, before starting the training, we put the GPS unit in the special belt position of the device and then we had to check the light before the start of the workout. At the end of the workout, we removed the GPS unit from the belt and entered the docking station to store information. Data storage was done by updated specialized AMS software (Gpsports Systems Pty Ltd, Majura, Australia)^17^. We set the GPS default to SPI IQ Absolutes throughout the season. The variables used in this study were as follows among the external loaded variables: 1. BL, considered the newest workload training, calculates the training load marker as well as the rate of training load (BL/min); 2. MPA calculates the average rate of energy consumed per second (W/kg) for the athlete according to the specifications entered in the device default (i.e. height and weight), and the previous report showed that it has high reliability of 3–5%^18,19^; 3. AccZ1 (< 2 m/s^2^); 4. AccZ2 (2 to 4 m/s^2^); 5. AccZ3 (> 4 m/s^2^); 6. DecZ1 (< − 2 m/s^2^); 7. DecZ2 (− 2 to − 4 m/s^2^); 8. DecZ3 (> − 4 m/s^2^^20^).

#### Calculating the training load

From the BL we obtained, respectively; wAW (Total BL during the week was considered); wCW (Total AW 3 weeks ago divided into three); wACWR (calculated by wAW ÷ wCW obtained that week), uncoupled method^21^ was used to reduce the re-porting error in this study. For this reason, in the first four weeks of the season, two variables (wCW and wACWR), were not reported in the study; wTM (calculated by wAW ÷ SD)^22^; wTS (calculated by wAW × wTM); Ultimately, for other accelerometer variables calculations by form the weekly in during a throughout the season for 48 weeks.

### Statistical analysis

Shapiro–Wilk test was used for normality, and Levene’s tests for homogeneity. The information was used for descriptive (mean and SD), and the information analysis was inferential tests. One-way ANOVA was used to find the differences between groups, and then the Bonferroni *post-hoc* test was used to detect the pairwise comparisons between different workload parameters and accelerometry variables by playing position. Cohen's d effect size with 95% CI was used for variables in this study. The Hopkins’ thresholds for Cohen’s *d* effect size statistics were used as follows: ≤ 0.2, trivial; > 0.2, small; > 0.6, moderate; > 1.2, large; > 2.0, very large; and > 4.0, nearly perfect^23^. Significant differences were considered for *p* ≤ 0.05. All statistical analyzes were considered by SPSS (version 25.0; IBM SPSS Inc, Chicago, IL) at a significance level of *p* ≤ 0.05. Prism software (GraphPad Software, Boston, USA) was used to draw the diagrams.

## Results

**Figure 1:**
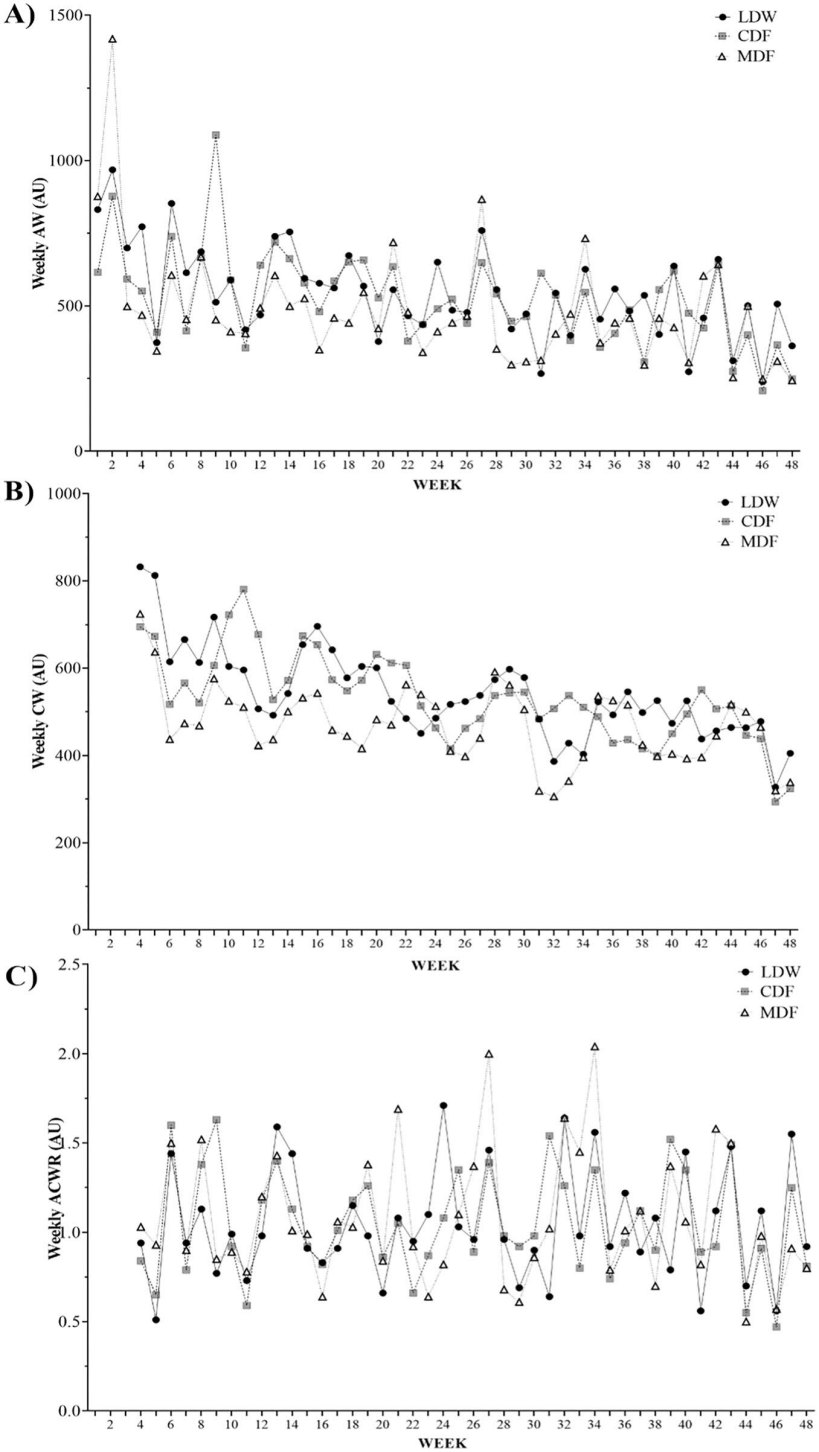
Overall vision of the (**A**) Weekly AW; (**B**) Weekly CW. (**C**) Weekly ACWR, variations across the full season by play position.

Figure 1 shows an overall vision of the wAW, wCW and wACWR, variations across the whole season by play position. Overall, the highest wAW occurred in weeks 27, 9 and 5 for LDW, CDF and MDF, respectively. The lowest wAW happened in week 46 for LDW and MDF, while CDF showed the lowest wAW in week 30 (Fig. 1A). Concerning wCW, the highest outcomes were observed in weeks 30, 11 and 6 for LDW, CDF and MDF, respectively. The lowest wCW were found in weeks 42, 48 and 26 for LDW, CDF and MDF, respectively (Fig. 1B). Besides, the highest wACWR happened in weeks 27, 22 and 5 for LDW, CDF and MDF, respectively. Coincidently, all player groups showed the lowest wACWR in week 30 (Fig. 1C).

**Figure 2:**
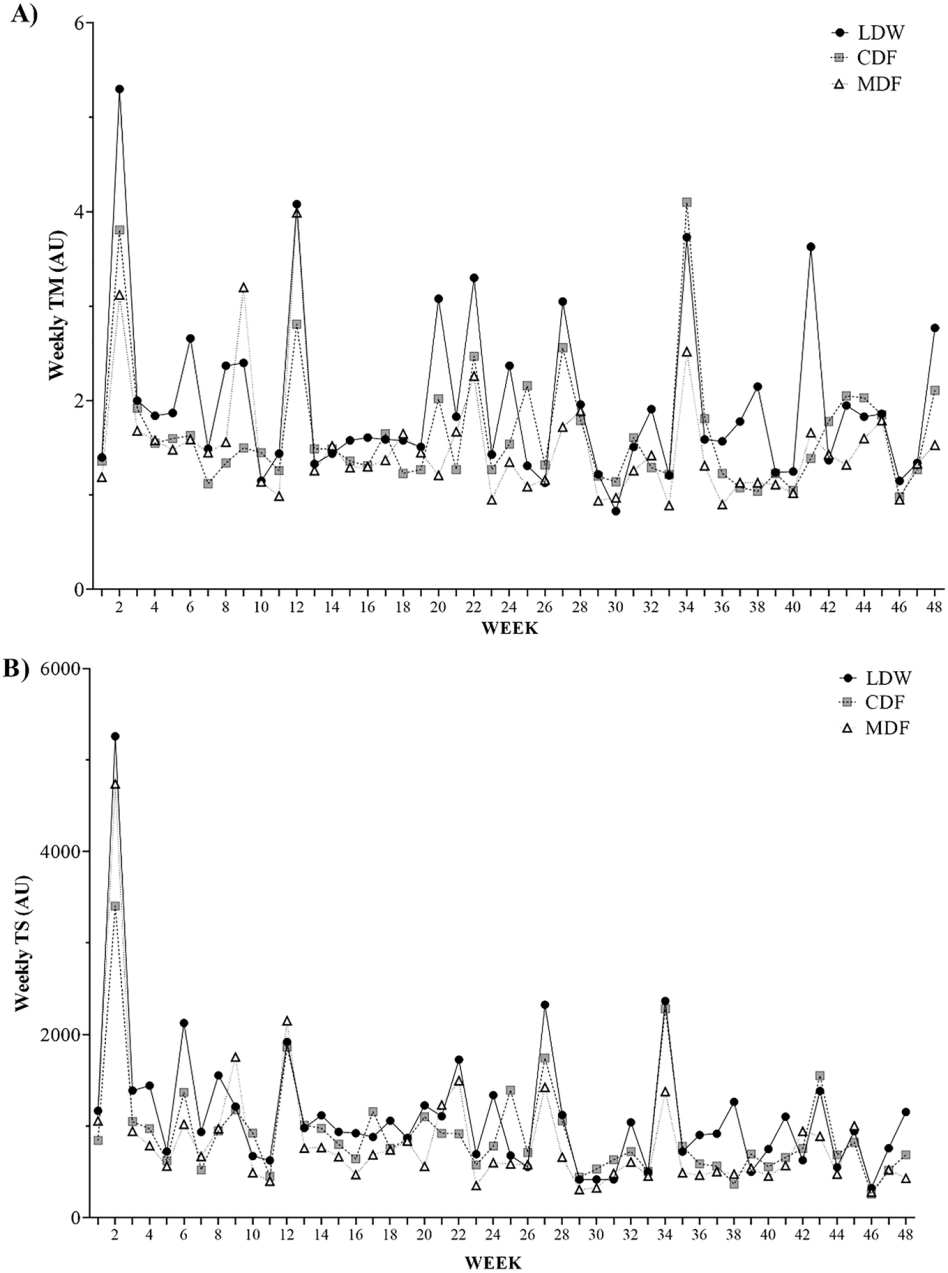
Overall vision of the (**A**) Weekly TM and (**B**) Weekly TS variations across the full season by play position.

Figure 2 displays the wTM and wTS variations across the full season by play position. The highest wTM occurred in weeks 2, 34 and 12 for LDW, CDF and MDF, respectively, while the lowest wTM was observed in weeks 29, 30 and 11 for LDW, CDF and MDF, respectively (Fig. 2A). Coincidently, the highest wTS happened in week 2 for all players groups. Moreover, the lowest wTS was observed in week 46 for LDW and CDF, while MDF showed the lowest wTS in week 29 (Fig. 2B).

Table 1 shows the between-group comparisons on wAW, wCW, wACWR, wTM, wTS, wMPA and wMPA/wAW for the full season. These comparisons were conducted using the full season average for every dependent variable, which was calculated from the weekly average of each one. Results revealed that there were no differences between player groups in any variables.

**Table 1:** Between-group differences in the full season weekly average for workload variables, training monotony and training strain.

	Mean (SD)	Comparative	Mean difference (95% CI)	P	Cohen's *d* (95% CI)	CV (%)
wAW (AU)	LDW: 547.24 (31.87)	LDW vs CDF	20.8 (− 67.7 to 109.4)	1.000	− 0.41 (− 1.44 to 0.61)	13.27
	CDF: 526.43 (60.57)	LDW vs MDF	73.8 (− 18.6 to 166.2)	0.148	− 1.03 (− 2.15 to 0.10)	
	MDF: 473.40 (96.46)	CDF vs MDF	53.0 (− 42.2 to 148.2)	0.476	− 0.62 (− 1.74 to 0.49)	
wCW (AU)	LDW: 541.32 (32.43)	LDW vs CDF	9.8 (− 79.7 to 99.4)	1.000	− 0.19 (− 1.20 to 0.83)	13.47
	CDF: 531.48 (64.14)	LDW vs MDF	72.0 (− 21.4 to 165.4)	0.171	− 1.01 (− 2.13 to 0.11)	
	MDF: 469.32 (95.20)	CDF vs MDF	62.2 (− 34.1 to 158.4)	0.317	− 0.72 (− 1.85 to 0.40)	
wACWR (AU)	LDW: 1.05 (0.04)	LDW vs CDF	− 0.001 (− 0.071 to 0.068)	1.000	0.02 (− 0.99 to 1.04)	4.61
	CDF: 1.05 (0.04)	LDW vs MDF	− 0.031 (− 0.104 to 0.041)	0.806	0.24 (− 0.82 to 1.30)	
	MDF: 1.06 (0.05)	CDF vs MDF	− 0.030 (− 0.105 to 0.045)	0.905	0.22 (− 0.87 to 1.31)	
wTM (AU)	LDW: 1.95 (0.56)	LDW vs CDF	0.31 (− 0.57 to 1.18)	1.000	− 0.48 (− 1.51 to 0.55)	37.32
	CDF: 1.65 (0.65)	LDW vs MDF	0.46 (− 0.45 to 1.38)	0.593	− 0.67 (− 1.76 to 0.41)	
	MDF: 1.49 (0.73)	CDF vs MDF	0.16 (− 0.79 to 1.10)	1.000	− 0.21 (− 1.30 to 0.88)	
wTS (AU)	LDW: 1120.15 (305.50)	LDW vs CDF	199.8 (− 347.6 to 747.1)	1.000	− 0.52 (− 1.55 to 0.51)	42.15
	CDF: 920.37 (418.21)	LDW vs MDF	327.4 (− 243.8 to 898.6)	0.443	− 0.78 (− 1.87 to 0.32)	
	MDF: 792.77 (487.42)	CDF vs MDF	127.6 (− 460.8 to 716.0)	1.000	− 0.26 (− 1.36 to 0.83)	
wMPA (W·kg^−1^)	LDW: 32.60 (5.51)	LDW vs CDF	3.3 (− 6.6 to 13.2)	1.000	− 0.47 (− 1.50 to 0.55)	25.14
	CDF: 29.29 (7.62)	LDW vs MDF	6.7 (− 3.7 to 17.1)	0.314	− 0.88 (− 1.99 to 0.23)	
	MDF: 25.90 (8.83)	CDF vs MDF	3.4 (− 7.3 to 14.1)	1.000	− 0.39 (− 1.49 to 0.71)	
wMPA/wAW	LDW: 0.060 (0.010)	LDW vs CDF	0.004 (− 0.009 to 0.018)	1.000	− 0.46 (− 1.49 to 0.56)	17.38
	CDF: 0.055 (0.009)	LDW vs MDF	0.007 (− 0.007 to 0.021)	0.593	− 0.63 (− 1.71 to 0.45)	
	MDF: 0.056 (0.010)	CDF vs MDF	0.002 (− 0.012 to 0.017)	1.000	− 0.21 (− 1.30 to 0.88)	

AU, arbitrary units; wAW, weekly average acute workload in AU; wCW, weekly average chronic workload in AU; wACWR, weekly average acute:chronic workload ratio in AU; wTM, weekly average training monotony in AU; wTS, weekly average training strain in AU; wMPA, weekly metabolic power average in watts per kilo-gram; wMPA/wAW, ratio between weekly metabolic power average and weekly average acute work-load; LDW, lateral defenders and wingers; CDF, central defenders and for-wards; and MDF, midfielders; P, P-value at alpha level 0.05; Cohen's *d* (95% CI), Cohen’s *d* effect size magnitude with 95% confidence interval; CV, coefficient of variations for overall team as percentage.

Between-group comparisons for derived-GPS variables of acceleration and deceleration in the full season were presented in Table 2. Overall, no significant differences were found between groups in any variables except for wAccZ2 and wAccZ3. Specifically, significantly higher wAccZ2 were observed in LDW compared to CDF (P = 0.006; *d* = 1.79) and MDF (P = 0.007; *d* = 1.79). However, no significant differences were observed when compared to CDF and MDF. Also, there were higher significant wAccZ3 in LDW compared to CDF (P = 0.003; *d* = 1.77) and MDF (P = 0.007; *d* = 2.13); no differences were observed when comparing CDF and MDF.

**Table 2:** Between-group differences for derived-GPS variables of acceleration and deceleration in the full season.

	Mean (SD)	Comparative	Mean difference (95% CI)	P	Cohen's *d* (95% CI)	CV (%)
wAccZ1 (< 2 m/s^2^)	LDW: 297.58 (34.36)	LDW vs CDF	25.8 (− 48.0 to 99.6)	1.000	− 0.54 (− 1.58 to 0.49)	20.00
	CDF: 271.75 (54.29)	LDW vs MDF	48.1 (− 28.9 to 125.1)	0.350	− 0.83 (− 1.93 to 0.27)	
	MDF: 249.47 (72.92)	CDF vs MDF	22.3 (− 57.0 to 101.6)	1.000	− 0.33 (− 1.42 to 0.77)	
wAccZ2 (2 to 4 m/s^2^)	LDW: 93.00 (9.84)	LDW vs CDF	10.1 (− 11.0 to 31.2)	0.673	− 0.78 (− 1.83 to 0.27)	19.33
	CDF: 82.93 (14.33)	LDW vs MDF	17.3 (− 4.7 to 39.4)	0.157	− 1.01 (− 2.13 to 0.11)	
	MDF: 75.65 (21.85)	CDF vs MDF	7.3 (− 15.4 to 30.0)	1.000	− 0.37 (− 1.47 to 0.73)	
wAccZ3 (> 4 m/s^2^)	LDW: 8.87 (1.38)	LDW vs CDF	0.89 (− 1.26 to 3.03)	0.870	− 0.63 (− 1.67 to 0.41)	21.18
	CDF: 7.98 (1.25)	LDW vs MDF	1.96 (− 0.28 to 4.20)	0.098	− 1.07 (− 2.20 to 0.06)	
	MDF: 6.91 (2.08)	CDF vs MDF	1.08 (− 1.23 to 3.38)	0.701	− 0.60 (− 1.71 to 0.52)	
wDecZ1 (> − 2 m/s^2^)	LDW: 145.33 (16.39)	LDW vs CDF	11.4 (− 46.0 to 23.3)	1.000	− 0.52 (− 1.55 to 0.51)	19.54
	CDF: 133.96 (24.77)	LDW vs MDF	25.2 (− 11.0 to 61.4)	0.248	− 0.91 (− 2.02 to 0.20)	
	MDF: 120.15 (34.76)	CDF vs MDF	13.8 (− 23.5 to 51.1)	1.000	− 0.43 (− 1.54 to 0.67)	
wDecZ2 (− 2 to − 4 m/s^2^)	LDW: 47.92 (5.64)	LDW vs CDF	5.5 (− 6.8 to 17.9)	0.759	− 0.80 (− 1.85 to 0.26)	22.12
	CDF: 42.38 (7.46)	LDW vs MDF	10.0 (− 2.9 to 22.9)	0.168	− 0.95 (− 2.07 to 0.16)	
	MDF: 37.93 (13.58)	CDF vs MDF	4.5 (− 8.8 to 17.8)	1.000	− 0.39 (− 1.49 to 0.71)	
wDecZ3 (< − 4 m/s^2^)	LDW: 13.51 (2.04)	LDW vs CDF	2.02 (− 1.66 to 5.69)	0.494	− 0.88 (− 1.94 to 0.18)	24.42
	CDF: 11.49 (2.27)	LDW vs MDF	3.28 (− 0.55 to 7.11)	0.110	− 1.06 (− 2.19 to 0.07)	
	MDF: 10.23 (3.75)	CDF vs MDF	1.26 (− 2.68 to 5.21)	1.000	− 0.39 (− 1.49 to 0.71)	
wAccZ1/wDecZ1	LDW: 2.05 (0.03)	LDW vs CDF	2.02 (− 1.66 to 5.69)	1.000	− 0.56 (− 1.59 to 0.48)	2.87
	CDF: 2.02 (0.05)	LDW vs MDF	3.28 (− 0.55 to 7.11)	0.996	0.47 (− 0.60 to 1.55)	
	MDF: 2.08 (0.09)	CDF vs MDF	1.26 (− 2.68 to 5.21)	0.348	0.71 (− 0.41 to 1.84)	
wAccZ2/wDecZ2	LDW: 1.94 (0.07)	LDW vs CDF	− 0.01 (− 0.20 to 0.16)	1.000	0.18 (− 0.84 to 1.20)	6.65
	CDF: 1.96 (0.09)	LDW vs MDF	− 0.09 (− 0.28 to 0.09)	0.622	− 0.59 (− 0.49 to 1.67)	
	MDF: 2.04 (0.22)	CDF vs MDF	− 0.08 (− 0.27 to 0.12)	0.900	0.46 (− 0.64 to 1.57)	
wAccZ3/wDecZ3	LDW: 0.66 (0.05)	LDW vs CDF	− 0.04 (− 0.11 to 0.05)	0.546	0.69 (− 0.36 to 1.73)	8.29
	CDF: 0.70 (0.07)	LDW vs MDF	− 0.03 (− 0.11 to 0.05)	1.000	− 0.58 (− 0.50 to 1.66)	
	MDF: 0.69 (0.05)	CDF vs MDF	0.01 (− 0.07 to 0.10)	1.000	− 0.20 (− 1.29 to 0.89)	

Notes: wAccZ1, weekly average of accelerations in zone 1 (< 2 m/s^2^) as number; wAccZ2, weekly average of accelerations in zone 2 (2 to 4 m/s^2^) as number; wAccZ3, weekly average of accelerations in zone 3 (< 4 m/s^2^) as number; wDecZ1, weekly average of decelerations in zone 1 (< − 2 m/s^2^) as number; wDecZ2, weekly average of decelerations in zone 2 (− 2 to − 4 m/s^2^) as number; wDecZ3, weekly average of decelerations in zone 3 (< − 4 m/s^2^) as number; wAccZ1/wDecZ1, ratio between weekly accelerations and decelerations in zone 1; wAccZ2/wDecZ2, ratio between weekly accelerations and decelerations in zone 2; wAccZ3/wDecZ3, ratio between weekly accelerations and decelerations in zone 3; LDW, lateral defenders and wingers; CDF, central defenders and forwards; and MDF, midfielders; P, P-value at alpha level 0.05; Cohen's *d* (95% CI), Cohen’s *d* effect size magnitude with 95% confidence interval; CV, coefficient of variations for overall team as percentage.

## Discussion

We aimed to analyze the weekly training loads throughout an entire season in a professional soccer team and also to compare these loads between players from different positions. Our results revealed that the weekly training loads varied throughout the season, and peaks of ACWR, training monotony, and training strain were more frequent in the pre-season and the midseason. However, no significant changes were observed between positions for all dependent variables. Therefore, we rejected our main *hypothesis*.

In general, the results of the present study showed that players from different positions were exposed to similar external loads throughout the season. Therefore, we expected higher weekly training loads for external players compared to the other playing positions because these players have shown the highest physical demand^14,15^ in official matches^14,15^. Also, when analyzing training contents, players from different positions showed different responses, even when the training scenario was the same^24,25^. The matches may represent the highest load within the training process and play a major role in biochemical and neuromuscular responses related to fatigue^7,26,27^. It is possible that coaches adopted tapering strategies to decrease the other training loads for these players, to reduce the stress and maximize performance, as suggested in previous studies^28^. This reduction probably compensated for the higher match load performed by external players leading to similar weekly loads among playing positions. Nevertheless, since we did not distinguish match-related load from the load imposed by other training activities, this hypothesis could not be confirmed.

The load monitoring over the whole season showed an oscillatory behavior of the external loads and a drastic increase in many variables between weeks 26 and 34. The congested fixture observed in many teams during the season highlights the importance of load monitoring and illustrates the difficulty of controlling the weekly loads over the competitive period. These sudden increases in the external loads require adequate strategists to reduce the injury risk, such as tapering^28^. The individualization of these strategies is also important to match the needs of each player, including the specificities related to playing position. Finally, the wACWR ranged between 1.05 and 1.08 arbitrary units for all playing positions, falling within the zone recommended to prevent injuries and reach positive adaptations^29^. We also observed recommended values of training monotony^11^ (below 2.00) in all playing positions. Both ACWR and training monotony indicate similar information when they control the training loads in soccer. However, we must be aware of the controversial aspects of ACWR^30^, such as the lack of a proper causal effect between ACWR and the injury rate. Therefore, future studies should seek clarification of these issues and comprehend the possible relationship between these variables.

This study provides an example of load distribution over the weeks of an entire season in professional soccer and highlights the importance of monitoring the external load throughout the season. Nevertheless, the participants of the present study were players from one professional team, which requires further investigation into the impact of playing positions on weekly training loads in different contexts or teams. Furthermore, we neither evaluate the training load of each day of the week, which could reveal the impact of each training activity (match, recovery, strength and conditioning, technical, tactical, others) on the weekly loads. Finally, it remains unclear whether the external load changes impact the players’ physiological responses and injury rate in soccer since most studies on load monitoring in soccer comprise only short periods. Providing further validity of the variables investigated in this study (such as ACWR, metabolic power, training monotony and training strain) would help sports scientists better understand the impact of load distribution over the season on players’ responses.

## Conclusions

More frequent peaks of ACWR, training monotony, and training strain were found in pre and mid season Moreover, despite an oscillatory training load dynamic was observed, these changes across the time are not position-dependent, as players from different positions showed similar training loads over the whole period. The absence of significant differences between positions could be related to the use of tapering strategies to increase sports performance in soccer competitions.

## Data Availability

The study results are presented clearly, honestly and without fabrication, falsification, or inappropriate data manipulation. All data are fully available upon email request to the corresponding author.
